# Optimal radiotherapy strategy for primary or recurrent fibromatosis and long-term results

**DOI:** 10.1371/journal.pone.0198134

**Published:** 2018-05-29

**Authors:** Seo Hee Choi, Hong In Yoon, Seung Hyun Kim, Sang Kyum Kim, Kyoo-Ho Shin, Chang-Ok Suh

**Affiliations:** 1 Department of Radiation Oncology, Yonsei Cancer Center, Yonsei University College of Medicine, Seoul, South Korea; 2 Department of Orthopedic Surgery, Yonsei Cancer Center, Yonsei University College of Medicine, Seoul, South Korea; 3 Department of Pathology, Yonsei Cancer Center, Yonsei University College of Medicine, Seoul, South Korea; University of Nebraska Medical Center, UNITED STATES

## Abstract

**Purpose:**

Although locally invasive or recurrent fibromatosis is primarily treated with surgery, radiotherapy (RT) produces local control for recurrent/unresectable tumors or those with positive surgical margins. Herein, we describe our updated institutional experience with RT to treat fibromatosis.

**Methods:**

Forty-seven patients with fibromatosis received RT between 1990 and 2015, and were followed for ≥12 months. Eight patients received RT for gross tumors, and 39 received postoperative RT after single/multiple prior surgeries. A median dose of 54 Gy was prescribed for definitive RT; 48.6, 50.4, and 54 Gy were prescribed for R0, R1, and R2 resected tumors, respectively. Recurrences were classified as in-field, marginal, or out-field. Prognostic factors were also evaluated.

**Results:**

Seven recurrences were noted, including 2 in-field, 4 marginal, and 1 out-field, after a median follow-up of 60 months. In-field recurrences occurred in 1 patient who received 40.5 Gy of salvage RT after postoperative recurrence and another who received 45 Gy for R1 resection after multiple prior operations. All marginal failures were due to insufficient clinical target volume (CTV) margins regardless of dose (3 with 45 Gy and 1 with 54 Gy). On multivariate analysis, a CTV margin ≥5 cm and dose >45 Gy were significant predictors of non-recurrence (p = 0.039 and 0.049, respectively). Subgroup analysis showed that patients with both an CTV margin ≥5 cm and a dose >45 Gy showed a favorable outcome.

**Conclusions:**

RT is a valuable option for treating aggressive fibromatosis; doses ≥45 Gy and a large field produce optimal results. For in-field control, a higher dose is more necessary for gross residual tumors than for totally excised lesions.

## Introduction

Aggressive fibromatosis (i.e., a desmoid tumor) is histologically benign but often shows aggressive features [1–3], as it can infiltrate adjacent tissues and cause local symptoms. The management of fibromatosis remains controversial, and the optimal treatment policy is still unclear [4]. Despite recent clinical guidelines [5–7], the rarity of fibromatosis and its complex behavior have rendered it difficult to establish universal treatment guidelines.

Surgery was once the primary treatment for aggressive fibromatosis. However, physicians have tended to avoid such interventions owing to high recurrence rates, possible iatrogenic morbidity, and a better understanding of a tumor’s natural history. Several predictors of tumor recurrence have been discovered; these have further stressed the need for adjuvant treatment in patients deemed to be high-risk [8]. To that end, radiotherapy (RT) has been suggested for patients with positive margins, recurrent lesions, or unresectable tumors. Although an optimal adjuvant RT protocol has yet to be adopted, many groups have reported long-term local control rates of 70–93% with a sufficient RT dose to the gross tumor.

There are no guidelines regarding the optimal radiation dose and extent of radiation volume for treating fibromatosis. Although several retrospective studies showed significantly more favorable local control rates at higher doses (50–60 Gy) [9–12], the dose-response relationship in patients with fibromatosis remains unclear. Moreover, since previous studies did not use unified RT fields, determining the exact dose-response relationship is difficult. In our previous series [13], we showed that aggressive fibromatosis with microscopic or gross residual disease could be well controlled with a moderate dose only if the field was adequate. The extent of coverage has also been shown to depend on several factors such as tumor size, number of prior recurrences, extent of surgery, and position of the tumor within a path of least resistance [14]. In previous studies, including ours, a generous surgical margin of 5–10 cm was generally recommended [11,12,15,16]. We suggested a margin ≥5 cm, regardless of adjacent normal tissue barriers, based on our data on marginal failures [13].

As a follow-up to our recent study, we present herein our updated experience and the long-term outcomes of additional patients with aggressive fibromatosis who received definitive or postoperative RT. We also offer new perspectives on RT doses and fields in patients with fibromatosis.

## Materials and methods

### Patient selection

We identified 56 patients who were diagnosed with fibromatosis and received their first RT between January 1990 and December 2015 at Severance Hospital. One patient who could not complete the whole RT session, 2 who had other secondary malignancies, 3 without follow-up data, and 3 without sufficient follow-up (<12 months) were excluded. Therefore, 47 patients were included in this retrospective study. The institutional review board of Yonsei University approved this study (approval number 4-2017-1064). The patient records/information was anonymized and de-identified prior to analysis, and informed consent was not obtained from each participants.

### Treatment

The treatment plans at our institution are individualized based on the clinical symptoms; location, size, and growth of the tumor; potential for complete resection without significant morbidity; and the patient’s preference. During our study period, planning often involved a multidisciplinary team of physicians from different departments. Surgical removal was primarily considered for fibromatosis when a safe surgical resection was possible. RT was performed in some patients with gross or microscopic residual disease following surgery or for those with unresectable tumors. RT was also performed in an adjuvant setting after a histologically complete resection, especially in patients with a recurrent tumor following surgery.

RT was administered using 4–10 MV X-rays, and 2D- or 3D-conformal RT was selected for most patients. Before 2000, 2D RT was used, whereas 3D RT was primarily employed thereafter. Considering the treatment site and target conformity, intensity-modulated radiotherapy was considered in some patients. Fractionation schedules were 5 days/week, with a daily fraction of 1.8 or 2 Gy. If the irradiated area was within the abdomen or the field was too broad, a daily fraction of 1.5 Gy was also considered. In both definitive and postoperative RT, we planned to administer a total dose of ≥45 Gy unless an exception was noted. When RT could be performed adjacent to the bowel, we considered a lower radiation dose. Conversely, if a gross tumor remained, a higher dose of >45 Gy was prescribed. For RT volumes, the gross tumor volume was defined as the entire operative bed/gross tumor as measured through imaging studies before and after surgery and was also based on pathological and surgical findings. The clinical target volume (CTV) was defined as the gross tumor volume plus a generous margin (>1.5–2 cm) to encompass the same muscle compartment. If other critical organs were located nearby, we reduced the margin tightly. The planning target volume was defined as the CTV plus a 0.5–1 cm margin. Our institutional technique was described in detail previously [13].

### Response evaluation

Patients were considered to have achieved local control if there was no evidence of disease during follow-up after the first RT. Although there was no standardized evaluation scheme owing to the disease’s benign nature, local recurrences could be detected by the patients themselves, by primary care physicians, or at follow-up visits at our institution. Computed tomography (CT), magnetic resonance imaging, and/or biopsy were used to verify locally recurrent disease when clinically evident. To correct for limitations resulting from individualized follow-ups, we analyzed only those with follow-up periods >1 year.

Responses were defined according to the Response Evaluation Criteria in Solid Tumors (version 1.1), as follows: complete response (CR), partial response (PR), stable disease, and progressive disease (PD). Recurrent lesions in patients with PD were classified as in-field, marginal, or out-field after comparing the recurrence volumes and dose-volume histograms of their first RT. The recurrence volumes were contoured using the diagnostic X-ray/CT/magnetic resonance images taken at that time, followed by a manual rigid co-registration with the RT planning CT using bony landmarks and visible soft tissue structures in the immediate vicinity of the recurred lesion. The dose-volume histograms of the first RT were regenerated. Treatment failure was classified as “in-field” if ≥95% of the recurrence volume occurred within the volume receiving ≥95% of the prescribed radiation dose, and as “marginal” if the recurrence volume border crossed the 95% isodose and <95% of the relapse volume was located inside the volume receiving ≥95% of the radiation dose. The failure was classified as “out-field” if it was completely outside the 95% isodose of the target volume.

### Statistical analysis

Progression-free survival (PFS) was measured from the first day of RT to the date of recurrence/progression or the last follow-up date for patients who did not experience such events. Overall survival (OS) was estimated from the first day of RT to the date of last follow-up or death from any cause. PFS and OS rates were calculated using the Kaplan-Meier method. Prognostic impacts of clinical factors were analyzed using the log-rank test (categorical variables) or logistic regression analysis (continuous variables). To compare the differences in outcomes according to RT dose, the patients were divided into 2 groups (high-dose vs. low-dose) using the median value as a cutoff. To investigate difference according to the RT margin (i.e., the CTV margin), the patients were divided into 2 groups (<5 cm vs. ≥5 cm margins) as described previously [13]. Differences in characteristics between the 2 groups were compared using the chi-squared test. All analyses were performed with SPSS version 23.0 (IBM Inc., Armonk, NY, USA) and R version 3.4.0. (R Foundation for Statistical Computing, Vienna, Austria). A p-value of <0.05 was considered statistically significant.

## Results

### Patient characteristics

The median age of the 47 patients was 28 (range, 4–66) years; the male-to-female ratio was 1:2.5. Patient and treatment characteristics are shown in Table 1. The lower extremities were the most common tumor locations (26%). At the time of treatment, the median tumor size was 7.0 (range, 2.2–26.0) cm.

**Table 1:** Patients and treatment characteristics.

Patient characteristics		No.	%	Treatment characteristics		No.	%
Age (year)		Median 28		Surgery	Yes	39	83
		(4–66)			No	8	17
Sex	Male	13	28	RM_Status	R0	5	83
	Female	34	72		R1	21	13
Location	Neck	9	19		R2	13	33
	Axilla	2	4	Chemotherapy	Yes	2*	4
	Thorax	9	19		No	45	96
	Abdomen	2	4	RT modality	2D	25	53
	Pelvis	4	9		3D	21	45
	Upper extremity	6	13		IMRT	1	2
	Lower extremity	12	26	RT duration (days)		Median 40	
	Shoulder	3	6				
Tumor Size (cm)		Median 7.0		RT total dose (Gy)		Median 47.0	
		(2.2–26.0)				(39.6–64.8)	
	<7cm	22	47	RT fractional dose (Gy)		Median 1.8	
	≥7cm	25	53			(1.5–2.0)	

* Chemotherapy before RT was performed in two patients, and the regimens were Xeloda in one patient and prednimustine, adriamycin, vincristine (VAP) in one patient.

***Abbreviations***: RM, resection margin; RT, radiotherapy; IMRT, intensity-modulated radiotherapy.

**Fig 1:**
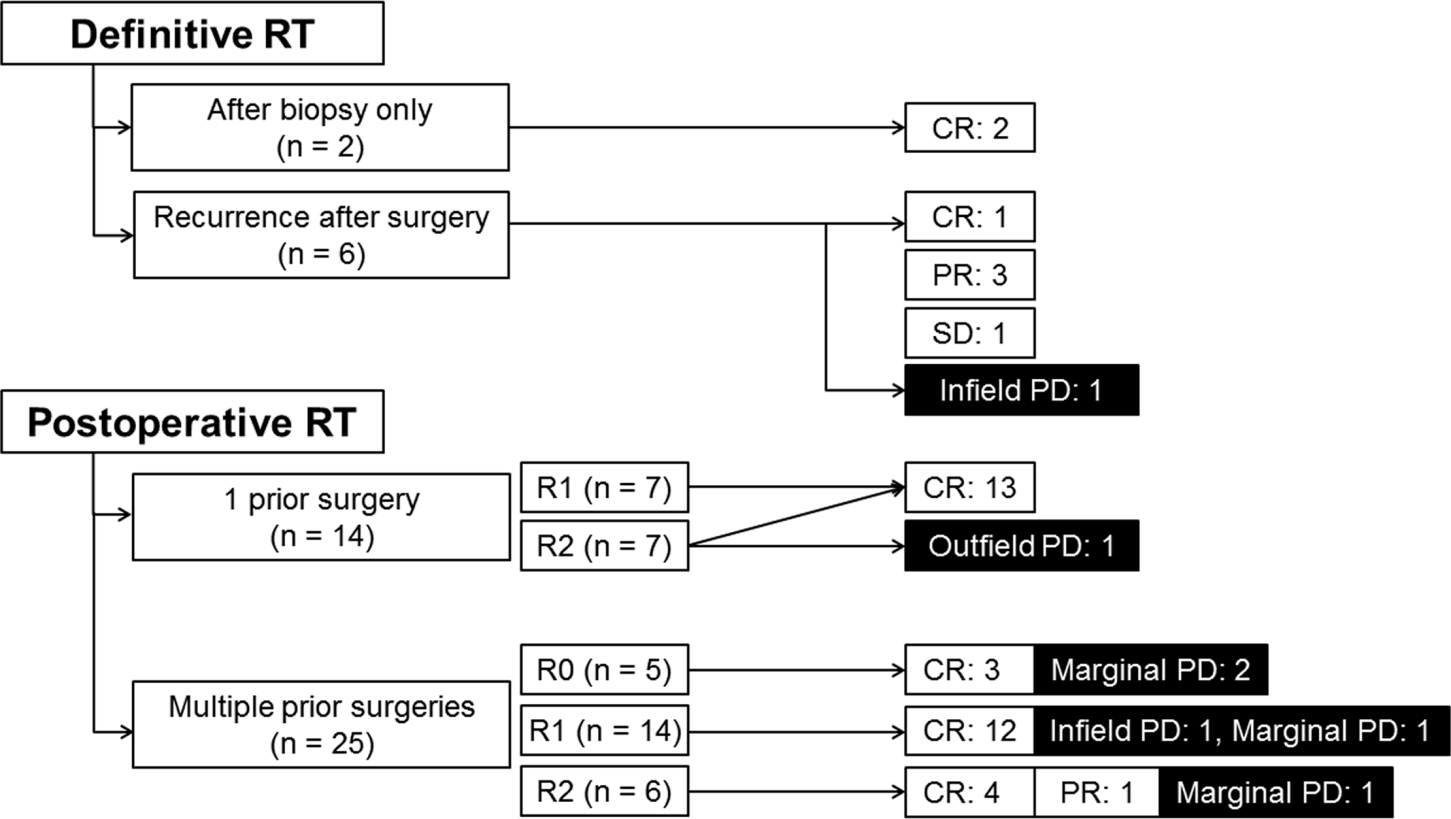
Treatment flowchart of 47 patients with fibromatosis. RT, radiotherapy; CR, complete response; PR, partial response; SD, stable disease; PD, progressive disease.

Eight patients received definitive RT for gross tumors as a single (n = 2) or salvage treatment (n = 6), while the remaining 39 received postoperative RT after single (n = 14) or multiple prior surgeries (n = 25). Among these 39 patients, 5 received RT after R0 resection, 21 after R1 resection, and 13 after R2 resection. Chemotherapy before RT was performed in 2 patients. After showing no response, chemotherapy was discontinued and RT was performed with or without surgical resection. The treatment flowchart is shown in Fig 1.

A median dose of 54.0 (range, 40.5–54.0) Gy was prescribed for definitive RT, and 50.4 (range, 39.6–64.8) Gy was prescribed for postoperative RT. Thirteen patients received 45 Gy while 5 received <45 Gy. Twenty-nine patients received >45 Gy, of whom 19 received ≥54 Gy. One patient received <40 Gy owing to a very young age (4 years) and the tumor’s location in the right infratemporal fossa. Median doses of 54, 50.4, and 48.6 Gy were administered for R2 (n = 13), R1 (n = 20), and R0 (n = 5) resected tumors, respectively. Intensity-modulated radiotherapy was administered to 1 patient who received 50 Gy of definitive RT for a large mass of approximately 8 cm on the left shoulder.

### Treatment response and prognostic factors

The median follow-up duration was 59 (range, 12–266) months. Two patients who received definitive RT as the initial treatment showed CR and no recurrence until the last follow-up (Fig 1). Meanwhile, of 6 patients who received salvage RT, 1 showed CR, 3 showed PR, 1 showed stable disease, and 1 showed in-field PD. Among patients with postoperative RT, CR and PR rates of 82% and 3%, respectively, were noted. One patient died of disease-related causes 150 months after RT. The 5-year PFS and OS rates were 87% and 100%, respectively. The outcomes of the 7 patients with PD are shown in Table 2. All patients underwent postoperative RT after repeat surgeries for repetitive recurrences, except for 1 who received salvage RT for gross recurrence after 2 prior surgeries. All received 40.5 or 45 Gy, except for 1 who received 54 Gy for an R2 resected lower extremity tumor and experienced marginal failure due to an insufficient CTV margin. In patients without recurrence, a median dose of 50.4 Gy for R0 or R1 resected tumors and 54 Gy for R2 resected tumors were prescribed.

**Table 2:** Detail of seven patients who underwent recurrences.

No.	Age (yr)	Sex	Tumor location	Tumor size (cm)	RT aim	No. of prior surgeries	RM status	RT dose (cGy)	CTV margin	Group	Recurrence type	PFS (mo)	Current status
1	37	F	Abdominal wall	3.5	Salvage	2		4050	<5cm	4	Infield	16	AWD
2	17	F	**Lower extremity**	10.0	Postoperative	2	**R2**	**5400**	<5cm	3	Marginal	63	NED
3	12	F	Upper extremity	9.0	Postoperative	2	R0	4500	**≥5cm**	2	Marginal	80	NED
4	30	F	Shoulder	18.0	Postoperative	2	R0	4500	<5cm	4	Marginal	8	DOD
5	21	F	**Lower extremity**	4.0	Postoperative	1	**R2**	4500	**≥5cm**	2	Outfield	51	AWD
6	15	F	**Lower extremity**	19.0	Postoperative	2	R1	4500	<5cm	4	Marginal	29	NED
7	8	M	**Lower extremity**	6.6	Postoperative	2	R1	4500	**≥5cm**	2	Infield	20	NED

***Abbreviations*:** RT, radiotherapy; RM, resection margin; CTV, clinical target volume; PFS, progression-free survival; AWD, alive with disease; NED, no evidence of disease; DOD, died of disease.

On univariate analysis, primary tumor location and RT dose were significantly associated with PFS (p = 0.027 and 0.035, respectively). Recurrence rates were higher in the abdomen and lower extremities. For postoperative RT, the 5-year PFS rates were not significantly influenced by resection margins. However, a CTV margin of ≥5 cm and an RT dose of >45 Gy were associated with significantly lower recurrence rates (p = 0.039 and 0.049, respectively), suggesting that both were clinically important factors for recurrences of any type (S1 Table).

#### Failure patterns and impact of CTV margin and dose

**Fig 2:**
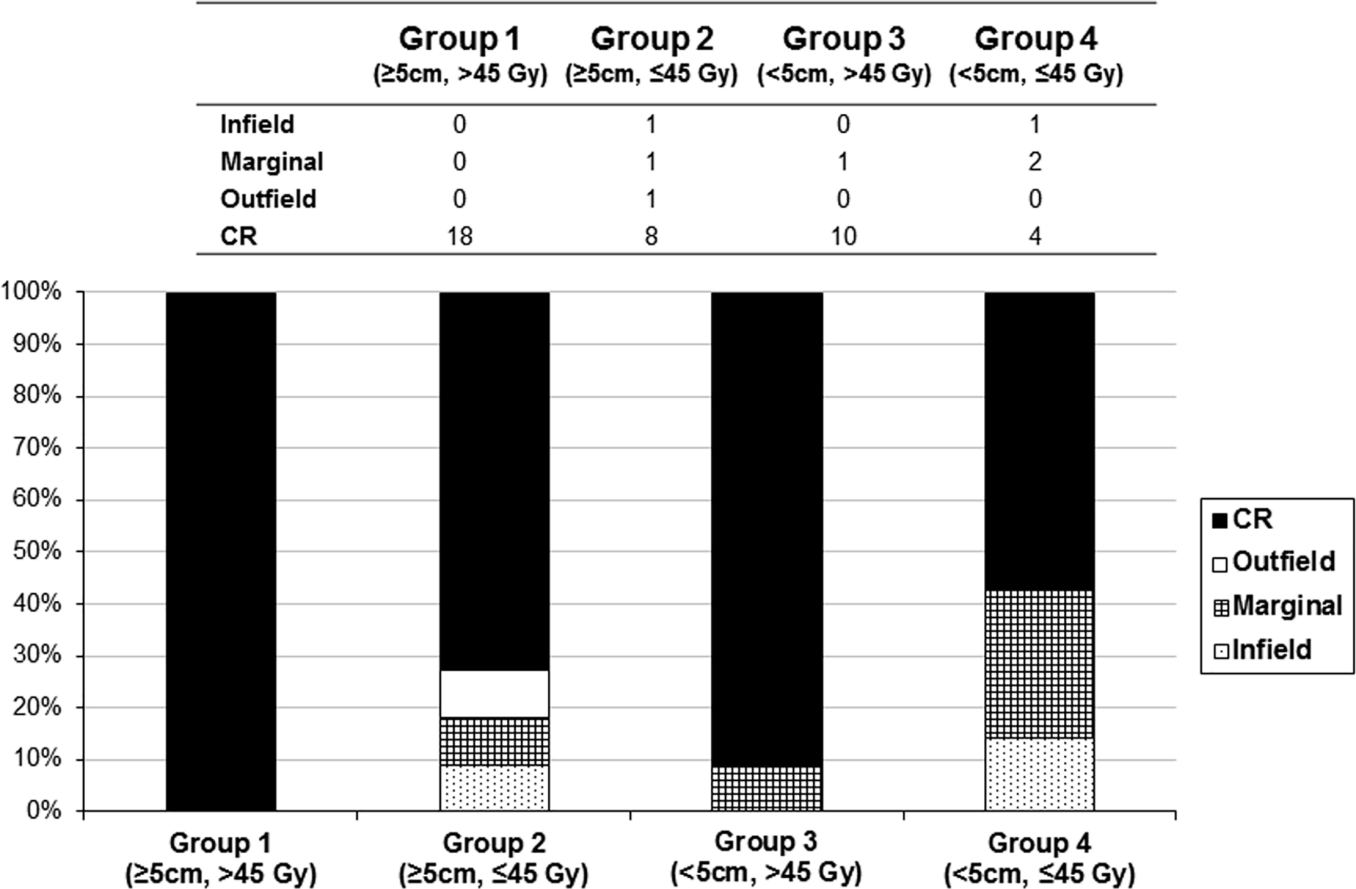
Patterns of failure among patients categorized into 4 groups according to radiotherapy dose and clinical target volume (CTV) margin. CR, complete response.

**Fig 3:**
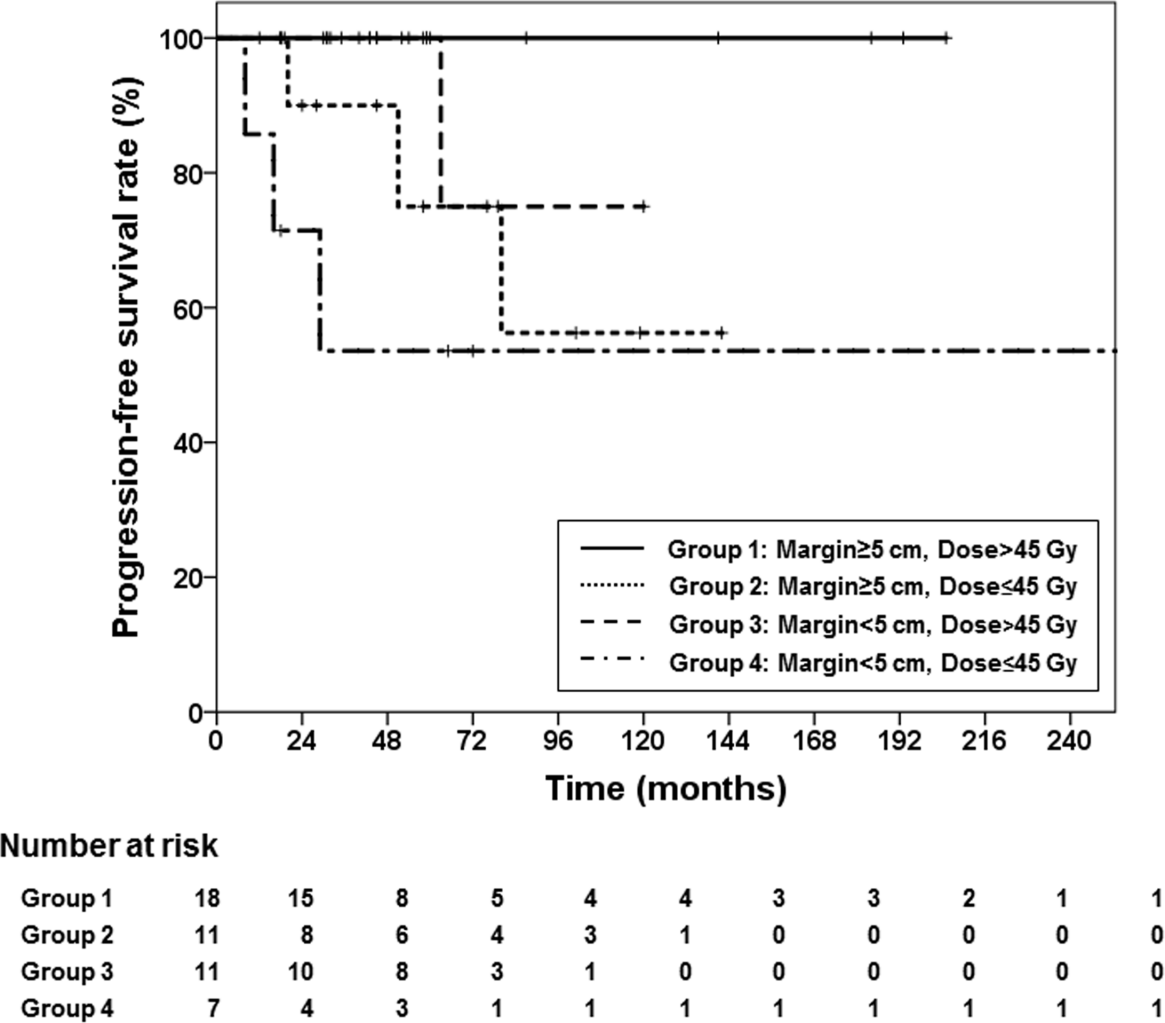
Progression-free survival among patients categorized into 4 groups according to radiotherapy dose and clinical target volume (CTV) margin.

**Fig 4:**
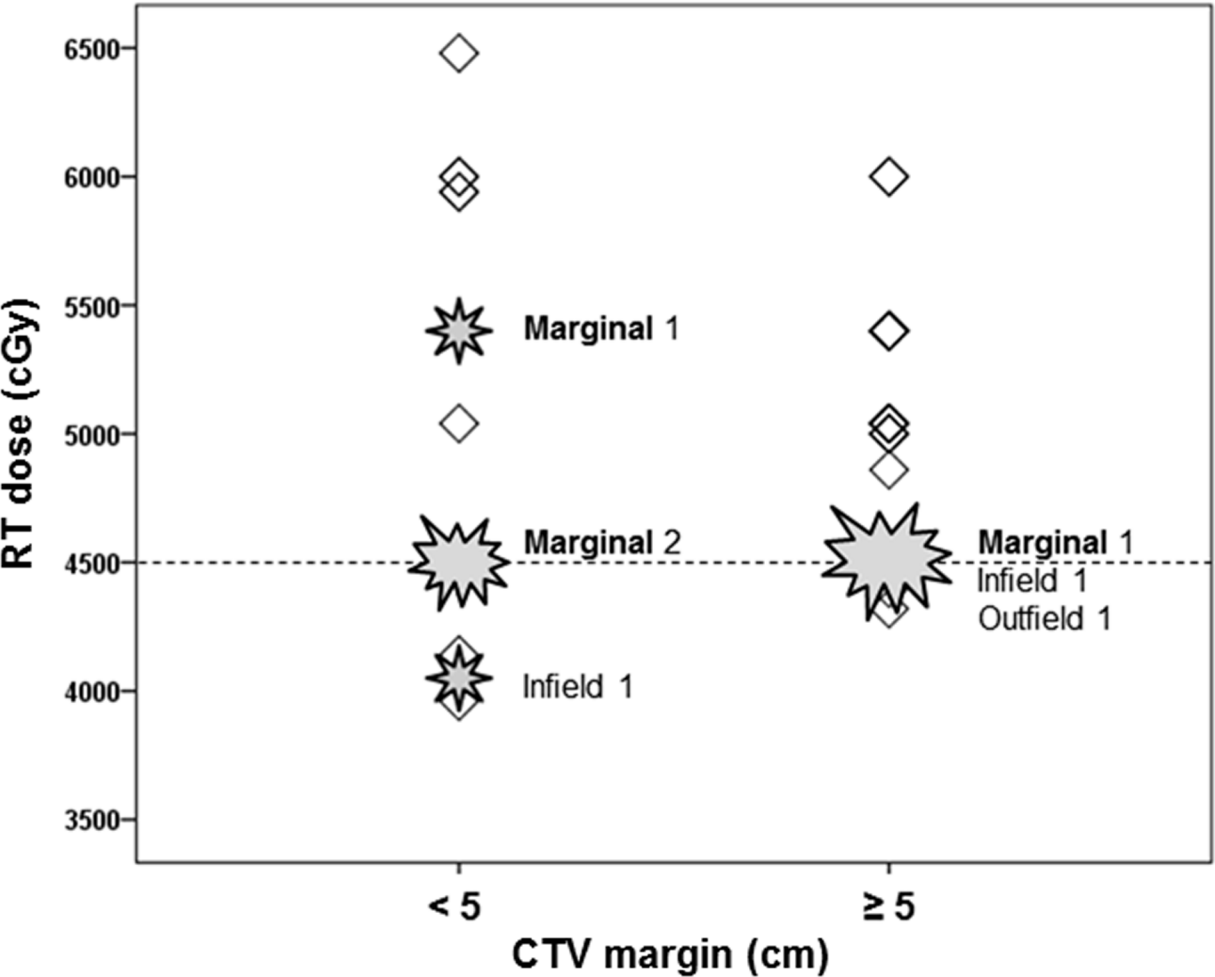
Scatter plots showing distributions according to radiotherapy dose and clinical target volume (CTV) margin. Stars represent patients with recurrence. The left large star represents 2 patients (at the same dose and field) together, and the right large star represents 3 patients together. RT, radiotherapy.

Although the RT dose was significantly associated with prognosis (hazard ratio, 0.998; 95% confidence interval, 0.996–1.000; p = 0.038) according to logistic regression (S1 Fig), the importance of the RT field was further emphasized when analyzing the specific failure pattern. To further investigate the differences in recurrence patterns as a function of CTV margin and dose, we categorized the patients into 4 groups as follows (Fig 2): Group 1 (margin ≥5 cm, dose >45 Gy), n = 18; Group 2 (margin ≥5 cm, dose ≤45 Gy), n = 11; Group 3 (margin <5 cm, dose >45 Gy), n = 11; and Group 4 (margin <5 cm, dose ≤45 Gy) n = 7. The numbers of recurrences in these groups were 0, 3, 1, and 3, respectively (Fig 2), while the 5-year PFS rates were 100%, 75%, 100%, and 54%, respectively (Fig 3). No recurrences occurred in group 1; however, marginal recurrence rates increased in group 3 (4 patients; margin, <5 cm). In-field recurrences were noted in group 2 (4 patients; RT dose, ≤45 Gy). The in-field was well-controlled at a high RT dose of ≥45 Gy in most patients (Fig 4); only marginal failures due to insufficient CTV margins occurred regardless of dose sufficiency.

No in-field failure occurred among patients who received >45 Gy (Table 2). Only 2 patients who received 40.5 Gy for gross recurrence (because of tumor multiplicity and tumor location close to the small bowel) and 45 Gy for an R1-resected tumor after repetitive recurrences experienced in-field recurrences. Only 1 out-field failure event occurred in a patient who received a large field of 45 Gy for an R2-resected tumor of the left calf; the recurrence occurred at the left foot after 51 months. Her disease was well controlled after 118 months following 50.4 Gy of salvage RT.

Marginal failures occurred in 4 patients, and were due to insufficient (<5 cm) CTV margins regardless of the RT dose. Two patients (Nos. 2 and 6) underwent postoperative RT for lower extremity tumors; however, relapse occurred at the upper area of the <5 cm CTV margins. The superior margin of the field in each patient extended to approximately 3 and 4 cm from the superior end of the CTV, respectively; CR was achieved after the first RT. The recurrent tumors were located at the superior margin of the initial radiation field after 63 and 29 months in these patients, respectively. Patient No. 2 had a well-controlled status after surgery and 45 Gy of repeat RT. Patient No. 6 also underwent surgery and 45 Gy of repeat RT; however, another recurrence in the second RT field was noted, which was well-controlled as of the last follow-up. Another patient (No. 4) received postoperative RT in the inferior portion of the left trapezius muscle after prior surgeries, and experienced recurrence at the medial margin of the RT field, along with the cervical and thoracic vertebrae, 9 months later. The remaining patient (No. 3) received 45 Gy after prior surgeries of the left humerus. The RT margin was >5 cm in the proximal and distal directions. However, considering a skin reaction near the axilla, we could not provide a margin of up to 5 cm diagonally, and recurrence occurred in this area after 80 months.

## Discussion

Our previous study [13] analyzed the treatment outcome of 24 patients who received RT between 1990 and 1998, and emphasized the importance of a wide RT field. The current study subsequently analyzed 47 patients who received RT during a longer period (1990–2015). Analysis of the patterns of failure revealed that a sufficient CTV margin remains critical. Moreover, we pointed out additional novel aspects related to the RT dose. In this updated analysis, a large radiation volume was deemed to be required regardless of the adjacent normal tissues. Moreover, a higher dose of 45 Gy may be required for gross residual tumors. At our institution, we prescribed a median dose of 54 Gy for definitive or postoperative RT for R2 resected tumors and 50.4 Gy for R1 resected tumors. With these doses, all but 2 of the 47 patients showed good in-field control rates, confirming that these doses were appropriate for such control.

RT can be an effective treatment option for aggressive fibromatosis and can be considered for both resected tumors with local recurrences and unresectable tumors as the sole treatment. Historically, RT was only used in cases of unresectable fibromatosis or in patients who declined surgery. More recently, RT has also been adopted as a primary treatment option in patients who experience progression after surgery. Several studies reported good long-term local control rates of 70–93% with RT [11,14,17–19]. Meanwhile, the evidence for adjuvant RT is insufficient. Nuyttens et al. [15] demonstrated that RT following surgery improved local control regardless of whether negative margins (94% vs. 72% with and without RT, respectively) or positive margins (75% vs. 41% with and without RT, respectively) were noted. However, conflicting studies [12,20–22] failed to show any benefit for adjuvant RT, leading to difficulty in establishing definitive guidelines. Most domestic institutions, including ours, lack a consensus for RT indications in fibromatosis, but RT is generally considered in patients with unresectable tumors, those who are likely to develop residual tumors after surgery, or those with a high local failure risk due to frequent recurrences [23,24]. Furthermore, a 100% in-field control rate in patients receiving definitive RT in this study, except for only 1 patient receiving 40.5 Gy, represents a high radiosensitivity for fibromatosis. Thus, future studies to widen RT indications are warranted.

Reliable predictors of an individual tumor’s natural history have been investigated by several groups. Young age, female sex, large tumor size, and tumors at the extremities [25] were independent negative predictors of recurrence-free survival after surgery. In particular, R2 resections of aggressive fibromatosis were strongly linked to postoperative recurrence [25,26]. However, conflicting results have also been reported [17,27], and a risk of local recurrence exists even after obtaining negative microscopic margins. However, we found no significant difference in prognosis according to resection margin status; only RT dose and field margin were significant prognostic factors. This difference may be due to the small sample size and/or the study’s retrospective nature, although RT itself can be a very strong prognostic factor. As in-field local recurrence is basically well controlled by RT, an appropriate RT field and dose are crucial. The PFS rate of 100% in group 1 of our current study (i.e., patients with a CTV margin ≥5 cm and dose >45 Gy) was observed (Fig 3); thus, the role of proper RT administration for any recurrences would be even more pronounced.

Although there were slight differences in the classifications of failure patterns, 82% of local failures in patients in a recent Finnish study [28] and 30% of local failures in American patients as reviewed by Nuytten et al. [15] were reported as marginal failures; therefore, adopting a sufficient RT margin is critical [14]. Despite a lack of consensus around an adequate RT margin, a wide safety margin of 5–10 cm has recently been recommended for fibromatosis [11,12,15,16]. The RT margin does not have to extend beyond the natural barriers of spread (i.e., fascial planes and bones); however, we strongly recommend a wide RT field coverage regardless of the adjacent normal tissue barriers. This updated study also strengthens our claim by reporting 2 more marginal failures for the same reason; 1 patient even died owing to an inadequate margin.

RT doses of 50–56 Gy have been suggested for fibromatosis [9,10], and even doses of ≥60 Gy were proposed for definitive RT. The MDACC study [29] demonstrated improved local control at >50 Gy for gross disease, but no improved outcome at >56 Gy. In a long-term follow-up study [12], a higher dose was not necessary in either the definitive or adjuvant settings, as radiation-related complications were significantly greater (>17%) when >56 Gy was administered. Moreover, Goy et al. [14] demonstrated better local control at >49.6 Gy, while Nuyttens et al. [15] found significantly improved in-field control at ≥50 Gy for gross tumors. In the multicenter phase II EORTC trial [30], investigators prescribed 56 Gy for inoperable tumors and reported a local control rate of 81.5%. However, in some studies, CR was achieved with a low dose of 35 Gy, while recurrences were detected despite doses >60 Gy [15]. Thus, the optimal dose remains uncertain, although a higher dose is still required for gross tumors. Our institution’s practice is to prescribe 45–50 Gy for fibromatosis, which is lower than in previous studies, based on our findings of locally well-controlled tumors with lower RT doses. No recurrences occurred with medians of 48 and 45 Gy for gross measurable tumors and microscopic tumors, respectively, in patients with an adequate RT field, and our updated results re-confirmed these findings. Generally, in-field failure is unlikely if ≥45 Gy is administered; only the RT field is of concern.

Although RT is effective in treating extra-mesenteric fibromatosis, more data for dose-response and long-term outcomes are needed. Ballo et al. [11,29] insisted that there was no dose-response relationship for patients treated with postoperative RT. Conversely, a study in Finland [28] recently demonstrated a significant dose-response relationship using definitive and postoperative RT. Our study also showed a significant dose-response relationship in all patients. However, recurrences of all types, and not necessarily in-field failures alone, were considered in these studies. Further validation is required by specifically analyzing in-field recurrences in larger patient cohorts.

Our study had several limitations. First, despite the long-term study period (approximately 25 years), we could not include many patients due to the low referral rate for RT combined with the rarity of fibromatosis. Second, there were differences between past and present pathological diagnostic criteria. Since 2005, beta-catenin staining has been essential for diagnosing fibromatosis, as it is a differentiating factor from other fibroblastic tumors [31,32]. Therefore, some unresponsive tumors previously diagnosed as fibromatosis might have been low-grade sarcomas that required more aggressive RT. Nevertheless, our study is meaningful given the difficulty of performing prospective studies on fibromatosis; there are only a few studies that demonstrate long-term outcomes with a large number of patients from a single institution.

In conclusion, RT for aggressive fibromatosis is a valuable option for achieving favorable long-term in-field control. Even gross residual fibromatosis can be well-controlled with a sufficient dose. However, establishing a wide radiation field considering all initial tumor extents is necessary, especially for tumors with repeated recurrences. Higher doses (50–54 Gy) should be considered for residual fibromatosis after surgery than for completely excised tumors.

## Supporting information

S1 Fig Relationship between radiotherapy (RT) dose (cGy) and any possibility of recurrence. (TIF)

S1 Table Univariate and multivariate analyses for progression-free survival. (DOCX)
