# Supplementary figures and images for: Optimal radiotherapy strategy for primary or recurrent fibromatosis and long-term results
Source: PLoS One. 2018 May 29;13(5):e0198134. doi: 10.1371/journal.pone.0198134 (PMC5973595; doi:10.1371/journal.pone.0198134)

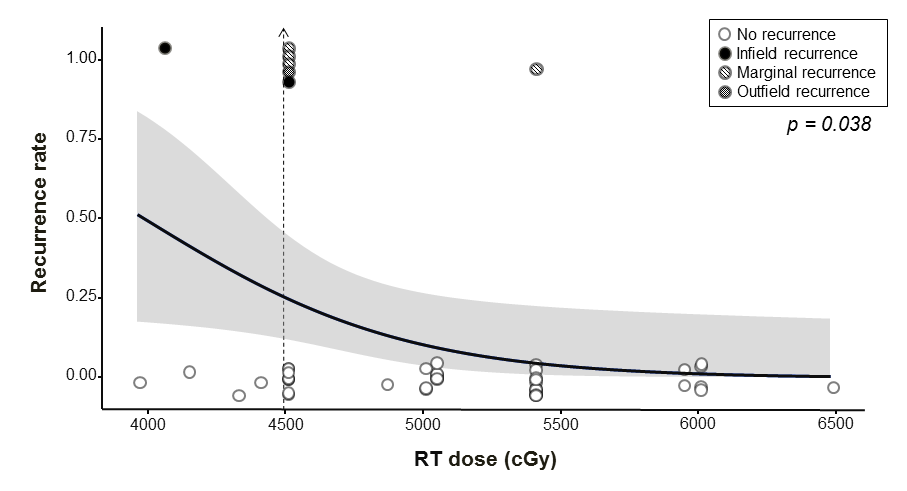

Supplement: S1 Fig — (TIF) [file pone.0198134.s001.tif]
