# Supplementary material for: Optimal radiotherapy strategy for primary or recurrent fibromatosis and long-term results
Source: PLoS One. 2018 May 29;13(5):e0198134. doi: 10.1371/journal.pone.0198134 (PMC5973595; doi:10.1371/journal.pone.0198134)
Supplement: S1 Table — (DOCX) [file pone.0198134.s002.docx]

**S1 Table.** Univariate and multivariate analyses for progression-free survival

|  |  | **PFS** | | | | | | |  |
| --- | --- | --- | --- | --- | --- | --- | --- | --- | --- |
|  |  | **Univariate analysis** | | |  | **Multivariate analysis** | | |  |
|  | **Variables** | **HR** | **95% CI** | **p-value** |  | **HR** | **95% CI** | **p-value** |  |
|  | Sex (Male vs. Female) | 3.017 | 0.360-25.314 | 0.309 |  |  |  |  |  |
|  | Location | 2.105 | 1.088-4.073 | 0.027 |  |  |  |  |  |
|  | Neck vs. Abdomen |  |  | 0.034 |  |  |  |  |  |
|  | Thorax vs. Abdomen |  |  | 0.034 |  |  |  |  |  |
|  | Thorax vs. Lower extremity |  |  | 0.038 |  |  |  |  |  |
|  | Neck, thorax vs. Abdomen vs. Others |  |  |  |  | 4.731 | 0.979-22.855 | 0.053 |  |
|  | Tumor size (≥7cm vs. <7cm) | 1.069 | 0.237-4.809 | 0.931 |  |  |  |  |  |
|  | Prior surgery | 0.690 | 0.080-5.936 | 0.736 |  |  |  |  |  |
|  | RM status | 0.636 | 0.184-2.196 | 0.474 |  |  |  |  |  |
|  | Chemotherapy | 0.046 | 0.000-40.999 | 0.695 |  |  |  |  |  |
|  | CTV margin (≥5cm vs. <5cm) | 0.417 | 0.091-1.914 | 0.260 |  | 0.174 | 0.033-0.917 | 0.039 |  |
|  | RT dose (>45 Gy vs. ≤45 Gy) | 0.102 | 0.012-0.854 | 0.035 |  | 0.118 | 0.014-0.995 | 0.049 |  |

***Abbreviations:*** RM, resection margin; RT, radiotherapy; CTV, clinical target volume; PFS, progression-free survival; HR, hazard ratio; CI, confidence interval.
